# A SNaPshot assay for the rapid and simple detection of hepatitis B virus genotypes

**DOI:** 10.3892/mmr.2014.2372

**Published:** 2014-07-14

**Authors:** GUOQI LAI, WENLU ZHANG, HONG TANG, TINGTING ZHAO, LIWEN WEI, YING TAO, ZENGCHAN WANG, AILONG HUANG

**Affiliations:** 1 Laboratory Animal Center, Chongqing Medical University, Chongqing 400016, P.R. China; 2 Key Laboratory of Molecular Biology on Infectious Diseases, Ministry of Education, Chongqing Medical University, Chongqing 400016, P.R. China

**Keywords:** SNaPshot assay, hepatitis B virus, genotypes

## Abstract

A simple technique for the identification of common genotypes of the hepatitis B virus (HBV) remains to be identified. The present study was conducted to establish such a methodology. Four plasmids of genotypes A-D and 123 clinical serum specimens of HBV-infected patients were genotyped. HBV genotypes would be detected successfully when the HBV genotype reached a viral load of 1 × 10^3^ copies/ml or the BC genotype mixed samples reached a 5% level. The lower limit of detection of HBV DNA in serum specimens was determined to be 2.14×10^2^ IU/ml. The assay sensitivity and specificity were 100% and the consistency was demonstrated to reach as high as 90.24 and 100% compared with that of the DNA sequencing and cloning. The frequencies of the genotypes B, C, BC, BD and BCD were found to be 65.0, 23.6, 7.3, 3.3 and 0.8%, respectively. The accuracy of detection of the mixed infections was also higher using the rapid and simple SNaPshot method compared with that achieved with the DNA sequencing methods. The results of the present study indicated that the SNaPshot technique accurately distinguishes the HBV genotypes A-D and is able to be readily applied as a monitoring tool in HBV prognosis and treatment.

## Introduction

Hepatitis B virus (HBV) infection is a severe worldwide health concern. HBV is a DNA virus with a rapid rate of mutation. Based on the heterogeneity of the HBV nucleotide sequence, the HBV strains are divided into eight genotypes, A to H, with a characteristic geographical distribution (1,2). For example, genotypes A and D are mainly found in Europe, Africa and the Americas (3), whereas genotypes B and C predominate in Asia (4). In the present study, genotype B is mainly observed in South China and genotype C is mainly found in North China (5–7). Numerous studies have indicated that the HBV genotypes are intrinsically linked to the severity of liver disease in acute and chronic HBV infections. B-type HBV is often associated with mild liver diseases (8,9), whereas C-type HBV typically results in severe liver diseases, including cirrhosis and liver cancer (10,11). The HBV genotypes have been proven to be responsive to antiviral therapy (12). Therefore, an assay for the identification of the HBV genotypes is of significance in improving the prediction of prognosis and the determination of the optimal treatment regimen for liver disease caused by HBV infection.

Conventional methods to determine the HBV genotype (13,14) are labor-intensive, inaccurate or expensive. For instance, INNO-LiPA is a simple method suitable for the analysis of small volumes; however, it is not cost-effective for efficient high-throughput analysis in a routine clinical diagnostic setting (15). HBV DNA sequencing is currently regarded as the gold standard for genotyping despite its low sensitivity for the evaluation of mixed infections and the requirement for interpretation of complex peak patterns.

In the present study, a SNaPshot assay was developed based on the polymerase chain reaction (PCR) amplification using fluorescent-marked terminators as well as capillary electrophoresis to simultaneously analyze the four common HBV genotypes (A-D). The SNaPshot technique was selected in order to effectively overcome the shortcomings of the other methods and for the development of an effective and feasible method of clinical analysis capable of providing improved prognosis prediction, treatment determination and patient monitoring.

## Materials and methods

### Samples and HBV DNA extraction

The samples of four HBV genotypes (A-D) plasmids and 123 clinical serum samples available in our laboratory were subjected to the multiplex SNaPshot assay. All reagents were originally obtained from Shanghai Sangon Inc. (Shanghai, China). Blood samples were drawn from 123 patients with an HBV-positive infection at The Second Affiliated Hospital of Chongqing Medical University (Chongqing, China) by venipuncture. The serum samples were obtained from whole blood which was allowed to clot and were then centrifuged and the serum samples were stored at −80°C prior to testing. The HBV DNA was extracted using a viral DNA extraction kit (Shanghai HuaShun Inc., Shanghai, China) according to the manufacturer’s instructions. All the procedures were in compliance with the Helsinki Declaration. The donors were included in the study following written informed consent. The study protocol was approved by the Ethics Committee of the Chongqing Medical University (Chongquing, China; reference number: CQMU 2010–25).

### HBV nested-PCR primers and SNaPshot probes design

Nested-PCR and SNaPshot probes designed for the present study are shown in Table I and II. The HBV nested-PCR primers were designed using Primer 3 software (http://frodo.wi.mit.edu) according to the base sequences of the conserved regions of various HBV genotypes (nt260–700 and nt310–610, respectively). Four pairs of SNaPshot probes used for the detection of HBV types A-D were designed to anneal with the sense strand immediately adjacent to the specific site. Each SNaPshot probe was synthesized with a different length of a poly(dT) tail to allow for separation of the SNaPshot products on the basis of size.

### HBV DNA PCR amplification

Amplification was performed in a volume of 25 μl containing 0.5 μl of 10 μM of each primer (P1 and P4), 12.5 μl master mix (Takara, Dalian, China), 10 ng template DNA dissolved in 0.5 μl solvent and 11 μl ddH_2_O. The PCR reaction conditions were as follows: 94°C for 4 min, 35 cycles of 94°C for 30 sec, 56°C for 40 sec and 72°C for 50 sec, and a final step at 72°C for 10 min. In total, 3 μl PCR product mixture was analyzed by 1.5% agarose gel electrophoresis. A volume of 0.5 μl negative PCR products was nested in P2 and P3 at PCR reaction conditions of 94°C for 4 min, 35 cycles of 94°C for 30 sec, 50°C for 30 sec and 72°C for 40 sec, followed by 72°C for 10 min.

### HBV PCR product purification

Subsequent to PCR, 15 μl PCR product mixture was treated for SNaPshot analysis. Treatment was conducted with 5 units *SAP* (shrimp alkaline phosphatase, Applied Biosystems, Grand Island, NY, USA) and two units *Exo* I (exonuclease I, Applied Biosystems) incubated at 37°C for 1 h, followed by incubation at 75°C for 15 min to remove excess deoxyribonucleotide triphosphates (dNTPs) and primers. The samples were then stored at 4°C. The PCR products for the sequencing reactions were purified further using ethanol/NaAc and stored at 4°C.

### SNaPshot analysis

SNaPshot assays were performed using a SNaPshot multiplex kit (Applied Biosystems). The reactions were performed in a total reaction volume of 10 μl containing 1 μl purified PCR product (5–10 ng), 5 μl SNaPshot ready multiplex mix (Applied Biosystems), 1 μl mixture of each of the four SNaPshot single base extension probe mixtures (final concentrations of each probe are shown in Table II) and 3 μl ddH_2_O. Thermal cycling was performed under the following conditions: 25 cycles of 10 sec each at 96°C, 10 sec at 50°C and 30 sec at 60°C. Labeled extension products were treated further with 1 unit *SAP* for 1 h at 37°C and 15 min at 75°C, then mixed with 0.5 μl extension products with 0.5 μl Genescan-120 LIZ size standard and 9 μl Hi-Di formamide (Applied Biosystems) and denatured at 95°C for 5 min. The products were then immediately placed in an ice bath for 5 min and relocated through a 3100 sequencing analyzer using POP-6 polymer electrophoresis (Orbita). The fluorescence signal was analyzed with Gene Mapper 3.5 software (Applied Biosystems). The samples were sequenced simultaneously with P1.

### Sequencing

The PCR products for sequencing reactions were electrophoresed on an ABI 3100 sequencing analyzer using POP-6 polymer (Orbita) with P1.

### Sensitivity analysis

Two approaches were used to determine the sensitivity of the SNaPshot method. The first one involved serial dilutions of the B-type plasmid DNA (1×10^0^–1×10^8^ copies/ml) in HBV-negative serum and the second one, 10 ng template DNA containing the B-type plasmid DNA at 0, 5, 10, 20, 50, 80, 90, 95 and 100% in the C-type plasmid.

### Specificity and accuracy analysis

The HBV genotypes, A-D, plasmids and 123 HBV-positive serum samples were assayed using the SNaPshot method and DNA direct sequencing. Where the results of SNaPshot and sequencing were inconsistent, the PCR products were cloned into the pMD18-T vector (Takara Bio, Inc., Shiga, Japan) according to manufacturer’s instructions and 20 clones of each sample were selected for further sequencing.

### Statistical analysis

The statistical package SPSS 11.5 (SPSS, Inc., Chicago, IL, USA) was employed for data analysis. P≤0.05 was considered to indicate a statistically significant difference.

## Results

### Strategies for HBV genotyping

**Figure 1:**
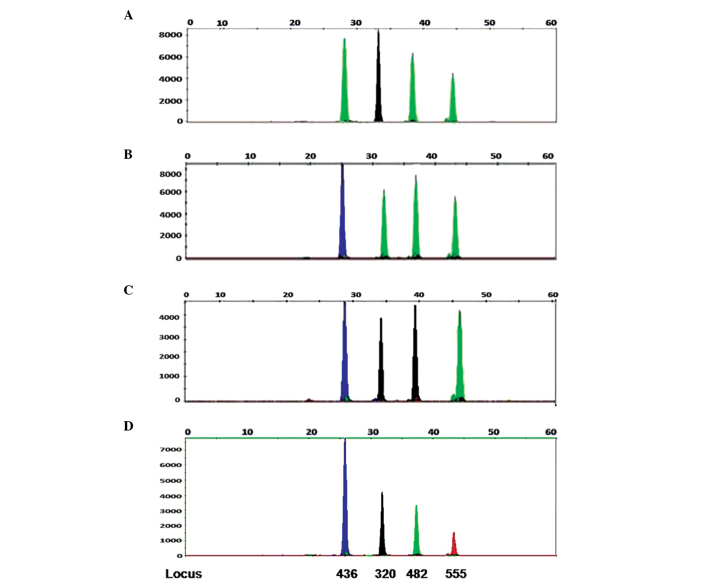
Strategies and specificity of the HBV A–D plasmids detected by the SNaPshot assay. SNaPshot results obtained from the analysis of the HBV plasmid. The HBV DNA templates were amplified by polymerase chain reaction, single base extension and electrophoresis. Probe 1 recognized the HBV locus 436, while probes 2, 3 and 4 recognized the HBV locus 320, 482 and 555, respectively. The x-axis represents the size (bp) of the probe pair with the incorporated nucleotides, while the y-axis corresponds to the relative fluorescence units of the peak. The probe mix included 1.5 μM probe A, 3.0 μM probe B, 0.75 μM probe C and 3.0 μM probe D. A–D correspond to the HBV genotypes, A–D, plasmids. Red refers to T, blue refers to G, green refers to A and black refers to C. Genotypes A, B, C and D correspond to ACAA, GAAA, GCCA and GCAT, respectively.

The strategies for detection of the HBV genotypes, A-D, are presented in Fig. 1. HBV DNA templates were amplified by PCR or nested-PCR, followed by a SNaPshot single base extension and electrophoresis. Probe 1 recognized the HBV locus 436, while probes 2, 3 and 4 recognized the HBV loci 320, 482 and 555, respectively. The single base extension of these four probes produced 22, 27, 33 and 39 bp fragments, respectively (Table II).

### HBV DNA amplification

HBV DNA PCR amplification appeared to be a major problem of the SNaPshot analysis. Two sets of primers for HBV PCR were designed to amplify the distinct fragments, 440 and 300 bp. Four DNA plasmids and 111 samples were effectively amplified using the outer primers. In total, 12 samples that were effectively amplified using the nested primers were subsequently quantified using a HBV nucleic acid detection kit (Zhongshan Da an Gene Co., Ltd., Guangzhou, China). The minimum concentration was 2.14×10^2^ IU/ml.

### SNaPshot analysis optimization

The probe length (21, 26, 32 and 38 bp), the concentration (1.5, 3.0, 0.75 and 3.0 μM; Table II), and the template concentration were optimized to ensure consistency in the height and position of the peaks representing the four types of extension products. A significant linear regression correlation with the peak (r=0.995) was identified at the DNA template concentrations ranging from 2.5–10 ng/μl.

### Specificity analysis

This novel multiplex SNaPshot method was validated by analysis of the four types of HBV genotypes (A-D) and the HBV-negative patient samples. The predicted results were obtained for the four plasmid types. Non-specific signals were observed and HBV-negative patient samples had no signal. The results indicated the detection of an HBV A-type for ACAA, a B-type for GAAA, a C-type for GCCA, and a D-type for GCAT. There were two different extension bases between the two different HBV types. Overall, this method demonstrated good specificity (Fig. 1).

### Sensitivity analysis

**Figure 2:**
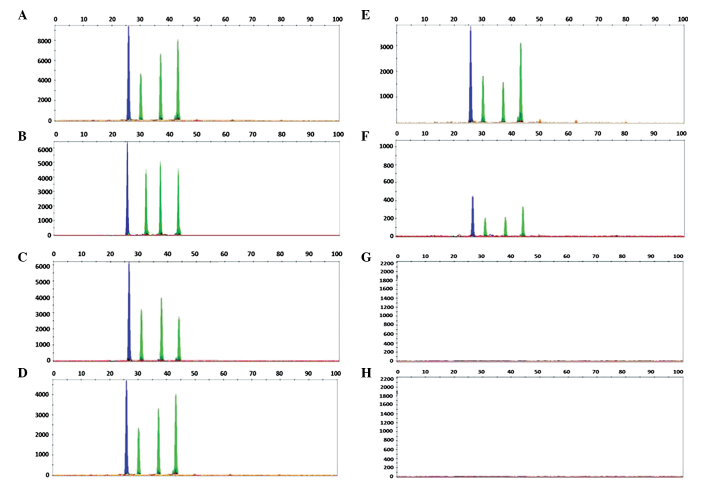
Sensitivity of the HBV B-type plasmid detected by the SNaPshot assay. (A–H), genotype B plasma containing DNA 1×10^8^; 1×10^7^; 1×10^6^; 1×10^5^; 1×10^4^; 1×10^3^ and 1×10^2^ copies/ml, respectively. 0 corresponds to the negative control. HBV, hepatitis B virus.

**Figure 3:**
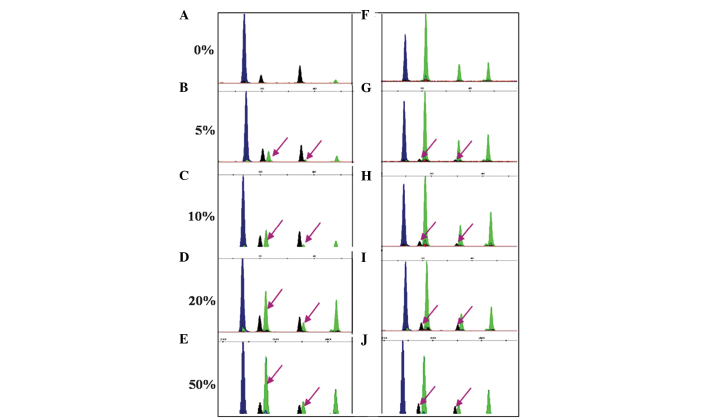
Sensitivity of the HBV BC mixed genotypes detected by the SNaPshot assay. (A–E) Proportion of the HBV B-type in HBV C-type infection from 0 to 50%. (F–J) Proportion of HBV C-type in HBV B-type infection from 0 to 50%. HBV, hepatitis B virus.

The sensitivity of the SNaPshot assay was assessed by analysis of the B-type plasmid diluted in HBV-negative serum and mixed samples containing the B-type with the C-type plasmids at varying concentrations or proportions. A correct analysis of the HBV genotype was achieved up to a viral load of 1×10^3^ copies/ml. Simultaneously, successful detection of BC mixed samples was achieved up to a 5% level. The results are shown in Fig. 2 and 3.

### Comparison of the SNaPshot assay and DNA sequencing

**Figure 4:**
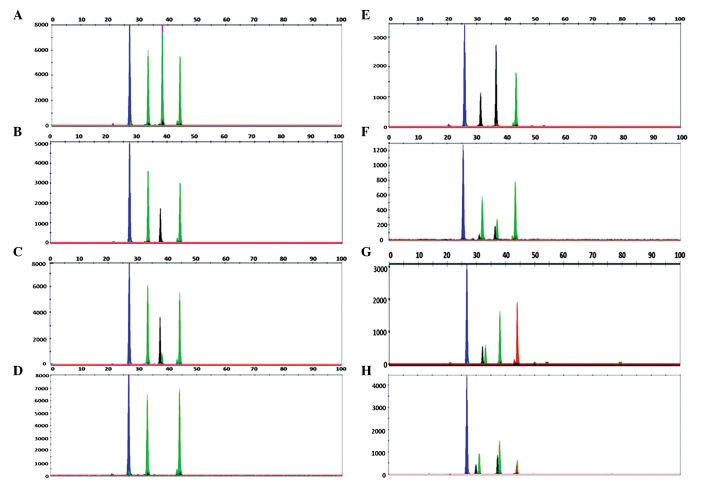
Results of HBV clinical samples detected by the SNaPshot assay. (A–D) genotype B; (E) genotype C; (F) genotype BC mixture; (G) genotype BD mixture and (H) genotype BCD mixture. HBV, hepatitis B virus.

The SNaPshot assay was evaluated using clinical serum samples from 123 patients identified with an HBV infection. All the samples were analyzed by the SNaPshot assay and DNA sequencing. Discrepancies in the results between the two methods were confirmed by subclone sequencing. Out of the 123 samples, 80 cases of B-type, 29 cases of C-type, nine cases of BC-type, four cases of BD mixed infection and one case of BCD-type were identified using the SNaPshot assay. In the detection process, four types of electrophoresis patterns were observed in the B-type samples, which included GAAA, GACA, GA (CA) A and GA-A. Fig. 4A–D show typical results from samples assayed by SNaPshot. DNA sequencing analysis revealed two base mutations in the GA-A samples differing in the no. 3 extension probe sequences. A comparison of the results obtained using the SNaPshot assay and DNA sequencing demonstrated 100% (123/123) sensitivity and validity of the SNaPshot assay. However, inconsistent results were obtained for 12 samples. Clone sequencing analysis of 20 randomly selected clones for each of these 12 samples was subsequently performed and the results were found to be fully consistent with the SNaPshot assay analysis (12/12; Table III; Fig. 4E–H). The consistency of the SNaPshot assay with DNA sequencing and clone sequencing assays was 90.24 (111/123) and 100% (12/12), respectively. The accuracy of the SNaPshot assay for detection of mixed HBV genotype infections was found to be equivalent to or higher than that of direct DNA sequencing [100% (14/14) vs. 14.29% (2/14); P=5.983×10^−6^ according to Fisher’s exact test].

## Discussion

In the present study, a SNaPshot assay was established for the simultaneous detection of the four most common HBV genotypes in clinical specimens. The methodology employed for this assay is also regarded as a short-sequencing technology. The reaction cocktails consisted of a polymerase, four fluorescent-labeled dNTPs and extension probes. The position of the nucleotide base at the termination site is associated with the genotype, and this is readily detectable by differential fluorescence analysis. As many as ten SNP polymorphic loci are able to be readily detected in a single run experiment.

The SNaPshot assay described in the present study utilized four colors of fluorescence labeling (green, red, blue and black) to allow single-peak fluorescence waveform identification of the four bases (A, T, G and C) with 3100 gene sequencing. The four HBV genotypes, A-D, were differentiated based on the simultaneous appearance of these four differential fluorescent labels: Green (A), red (T), blue (G) and black (C), for ACAA, GAAA, GCCA and GCAT, respectively. Mixed infections containing different HBV genotypes produced distinct bimodal distributions in separate locations of the electropherogram reflecting the number of different genotypes detected. Therefore, electropherograms generated from each species demonstrated that the genotypes examined, A-D, were able to be clearly identified and differentiated from one another using this novel SNaPshot assay.

The detection level of the SNaPshot assay based on nesting PCR was 1×10^3^ copies/ml. The detection level of BC-mixed infections was 5%. In the present study, 123 HBV isolates from clinical specimens (HBV DNA ≥ 2.14×10^2^ IU/ml) were analyzed using the multiplex SNaPshot assay. The test sensitivity of the standard plasmids and clinical samples was 100%. The incidence of B-type, C-type, BC-type, B-type and BCD-type was shown to be 65.04, 23.58, 7.32, 3.25 and 0.81%, respectively, using the SNaPshot assay. An analysis of the sequences of the GA-A electropherogram demonstrated that the no. 3 SNaPshot probes were not extended as a single base for the last base mutations (A-G). However, this did not significantly affect the determination of the HBV B-type. A comparison of the SNaPshot assay and DNA sequencing methods revealed that there were five cases of BC-, two cases of BD- and one case of BCD-type among the eight B-type samples; three cases of BC-type among the three C-type samples and one case of BD-type among the D-type samples all detected by DNA sequencing. The mixing ratio of the 12 clinical samples was lower than 20% and the DNA copies were below the test level.

Similar results were obtained by the SNaPshot and the cloning sequence analysis. The SNaPshot analysis demonstrated that the HBV B-genotype was predominant in the Chongqing region, followed by the HBV C-type. A small amount of the BC-, BD- and BCD-type infections were also identified in the Chongqing region, although the BCD-type infections were the least common. The results obtained using the SNaPshot assay were consistent with the DNA sequencing results and the DNA cloning sequence analysis in the detection of different HBV genotypes in the high-copy DNA samples (111/111) and the small number of mixed infection in the serum samples (12/12). The sensitivity of the assay was 100% (123/123) and the accuracy of detection of mixed types was higher compared with that of direct DNA sequencing [100% (14/14) vs. 14.29% (2/14); P=5.983×10^−6^].

The results of the present study demonstrate that the SNaPshot assay offers a rapid, robust and highly reliable alternative to traditional DNA sequencing methods for the identification of the HBV genotypes A-D. Although the complete DNA sequence can be detected by HBV genome sequencing, the test results are unclear, providing complex background signals with more numerous peaks and lower detection sensitivity for the mixed infections due to the presence of residual primers.

In conclusion, the present study showed that the SNaPshot method is able to be used for the simultaneous analysis of the four common HBV genotypes. The sensitivity and accuracy of this technique was shown to be higher with regard to a single product, simple peak shapes and no background noise compared with the sequencing method. The SNaPshot method is limited by its potential for the detection of known genotypes only. However, HBV genotype research is advancing and the gene sequence of the virus is now recognized worldwide. Thus, the present study provides the basis of a novel technique that is suitable for further development in clinical HBV genotyping.

## Figures and Tables

**Table I:** Primer sequences used in the HBV PCR protocol.

Name	Sequences
P1:HBV 260F	5′-CTCGTGGTGGACTTCTCTCA-3′
P2:HBV 310F	5′-GGCCAAAATTCGCAGTCCC-3′
P3:HBV 610R	5′-GATGATGGGATGGGAATACA-3′
P4:HBV 700R	5′-CGAACCACTGAACAAATGGCA-3′

HBV, hepatitis B virus; PCR, polymerase chain reaction.

**Table II:** Probe sequences and concentration used in the HBV SNaPshot protocol.

No.	Name	Genotype	Sequences	Concentration, μM	Production length, bp
1	Probe1	HBV A	5′-GCTGCTATGCCTCATCTTCTT-3′	1.5	22
2	Probe2	HBV B	5′-TTTTTTTGGCCAAAATTCGCAGTCCC-3′	3.0	27
3	Probe3	HBV C	5′-TTTTTTTTTTTATGTTGCCCGTTTGTCCTCTA-3′	0.75	33
4	Probe4	HBV D	5′-TTTTTTTTTTTTTTTTTTTTGTACAGCAACAGAGGGA-3′	3.0	39

HBV, hepatitis B virus.

**Table III:** Results of serum samples detection by the SNaPshot and sequencing assay.

	SNaPshot assay					
	
Tests and results	B-type	C-type	D-type	BC-type	BD-type	BCD-type
DNA sequencing, n=123						
B-type	80			5^a^	2^a^	1^a^
C-type		29		3^a^		
D-type					1^a^	
BC-type				1		
BD-type					1	
BCD-type						
Clone sequencing, n=12						
B-type						
C-type						
D-type						
BC-type				8		
BD-type					3	
BCD-type						1

The accuracy of the SNaPshot assay was compared with that of the DNA sequencing assay and clone sequencing assay. The consistency of the SNaPshot assay with DNA sequencing assay and clone sequencing assay was 90.24% (111/123) and 100% (12/12), respectively.

a Clone samples of 12 DNA types.
